# Supplementary figures and images for: Contrasting effects of Ksr2, an obesity gene, on trabecular bone volume and bone marrow adiposity
Source: eLife. 2022 Nov 7;11:e82810. doi: 10.7554/eLife.82810 (PMC9640193; doi:10.7554/eLife.82810)

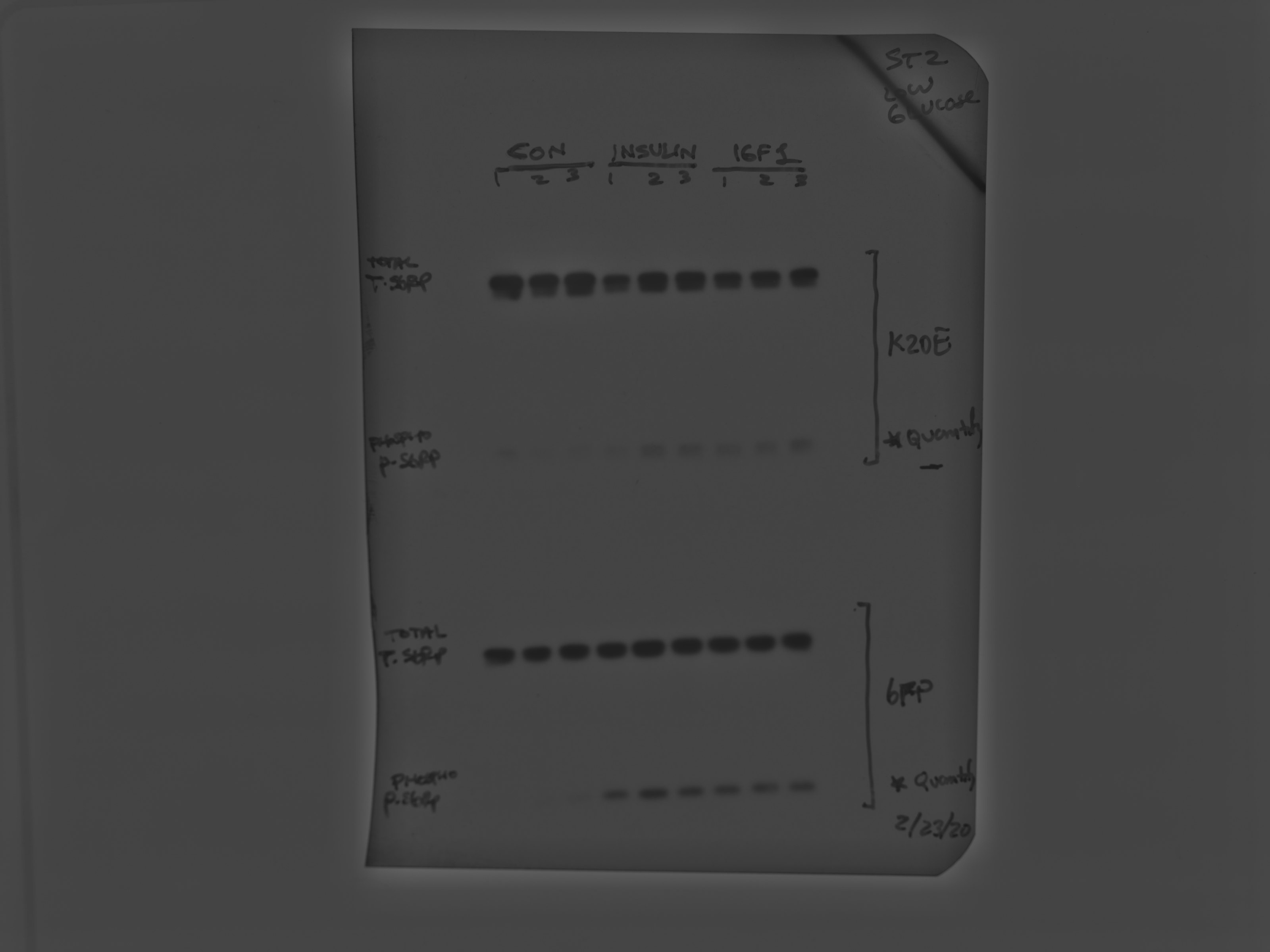

Supplement: Figure 8—source data 3. [file elife-82810-fig8-data3.zip › Figure 8- Source data 3.tif]

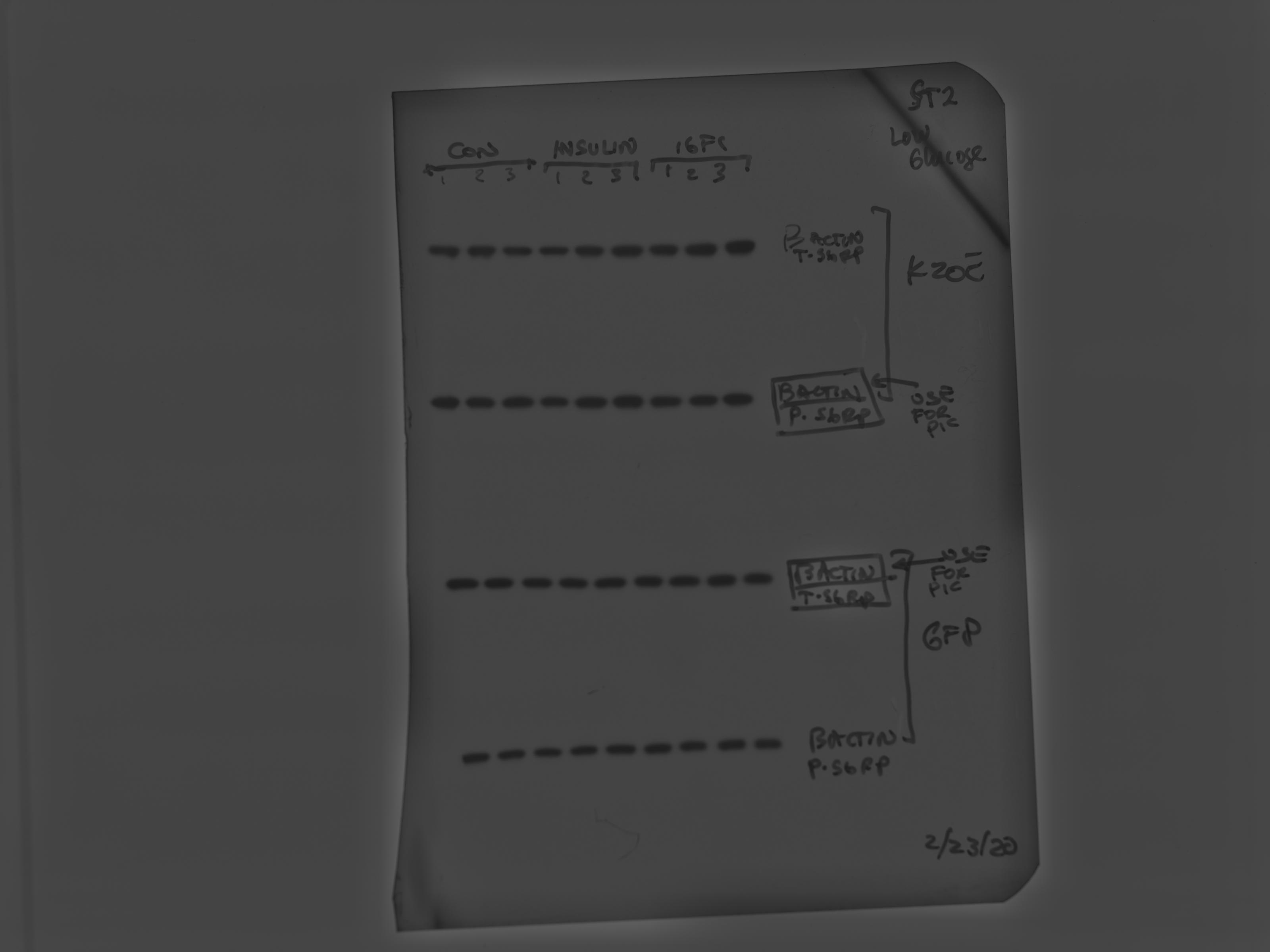

Supplement: Figure 8—source data 4. [file elife-82810-fig8-data4.zip › Figure 8- Source data 4.tif]
